# Extracellular vesicle-mediated targeting strategies for long-term health benefits in gestational diabetes

**DOI:** 10.1042/CS20220150

**Published:** 2023-08-31

**Authors:** Soumyalekshmi Nair, Valeska Ormazabal, Flavio Carrion, Aase Handberg, H David McIntyre, Carlos Salomon

**Affiliations:** 1 Translational Extracellular Vesicle in Obstetrics and Gynae-Oncology Group, UQ Centre for Clinical Research, Royal Brisbane and Women's Hospital, Faculty of Medicine, The University of Queensland, Australia; 2 Department of Pharmacology, Faculty of Biological Sciences, University of Concepcion, Concepción, Chile; 3 Departamento de Investigación, Postgrado y Educación Continua (DIPEC), Facultad de Ciencias de la Salud, Universidad del Alba, Santiago, Chile; 4 Department of Clinical Biochemistry, Aalborg University Hospital, Aalborg, Denmark; 5 Mater Research, Faculty of Medicine, University of Queensland, Mater Health, South Brisbane, Australia

**Keywords:** Extracellular Vesicles, insulin resistance, microRNA, Pregnancy, skeletal muscle

## Abstract

Extracellular vesicles (EVs) are critical mediators of cell communication, playing important roles in regulating molecular cross-talk between different metabolic tissues and influencing insulin sensitivity in both healthy and gestational diabetes mellitus (GDM) pregnancies. The ability of EVs to transfer molecular cargo between cells imbues them with potential as therapeutic agents. During pregnancy, the placenta assumes a vital role in metabolic regulation, with multiple mechanisms of placenta-mediated EV cross-talk serving as central components in GDM pathophysiology. This review focuses on the role of the placenta in the pathophysiology of GDM and explores the possibilities and prospects of targeting the placenta to address insulin resistance and placental dysfunction in GDM. Additionally, we propose the use of EVs as a novel method for targeted therapeutics in treating the dysfunctional placenta. The primary aim of this review is to comprehend the current status of EV targeting approaches and assess the potential application of these strategies in placental therapeutics, thereby delivering molecular cargo and improving maternal and fetal outcomes in GDM. We propose that EVs have the potential to revolutionize GDM management, offering hope for enhanced maternal–fetal health outcomes and more effective treatments.

## Introduction

Gestational diabetes mellitus (GDM) can be defined as glucose intolerance of a lesser degree than Type 1 and Type 2 diabetes and is first diagnosed during pregnancy [1]. Pregnancy is characterized by a decrease in insulin sensitivity, otherwise called insulin resistance which is the decreased biological response to insulin. Insulin resistance in pregnancy is beneficial as it limits the glucose utilization in maternal tissues and shunts the excess glucose in the maternal circulation to the fetus. Glucose being the major energy source of the fetus is utilized for the adequate growth and maintenance of fetal development. However, in mothers who develop GDM, maternal insulin production is insufficient to overcome insulin resistance and the mother develops hyperglycemia. As a result, the fetus is over nourished leading to serious metabolic dysregulation. GDM is the most common medical complication of pregnancy and leads to short- and long-term consequences for both mother and offspring.

The placenta secretes a plethora of molecules into the maternal circulation which are crucial to the regulation of maternal metabolism and development of insulin resistance in pregnancy. The placenta has intriguing roles in the development of excessive insulin resistance and pathophysiology of GDM [2]. This is evident from the fact that within days of delivery of the placenta, there is a substantial increase in maternal insulin sensitivity and the hyperglycemia of GDM generally resolves [3]. In addition, the metabolic changes associated with GDM, primarily maternal hyperglycemia and excess fatty acids and amino acids in maternal circulation adversely affect the placental nutrient transport. Exposure of the fetus to altered metabolite concentrations leads to serious short- and long-term effects in GDM offspring [4]. The placenta undergoes structural and biochemical changes to buffer the exaggerated insulin resistance in GDM and minimize the adverse effects on the fetus. Hence, the placenta is a contributor to the development of GDM, and the metabolic derangement in GDM consequently causes placental dysregulation.

The pivotal role of the placenta in GDM opens opportunities for targeting the placenta as a therapeutic option to improve maternal and fetal outcomes. Placenta targeting approaches have been reported as therapeutic options in pregnancy complications such as preeclampsia and fetal growth restriction [5–10]. These targeting approaches have used viral [5,8,10] and nonviral nanoparticles such as liposomes [6,7,11] as delivery vehicles for transferring chemical compounds and biomolecules to the placenta. Recent evidence shows EVs as ideal platforms for engineering therapeutic vehicles and delivering biomolecules in various biological conditions [12,13].

Extracellular vesicles (EVs) are membrane-derived vesicles, playing key roles in cell-to-cell communication and conveying molecular signals to cells at local and distant locations [14,15]. The finding that EVs can transfer molecular cargo to various cells has opened up novel opportunities to utilize EVs as therapeutic tools to deliver bio-active molecules or drugs to target cells [16–19]. Being a nanoparticle of biological origin, EVs have favorable pharmacokinetic and biodistribution profiles compared with nonbiological nanoparticles such as liposomes [20]. Moreover, engineering donor cells to produce EVs with desirable chemical and physical modifications using state-of-the-art techniques has tremendously improved the targeting strategies in EV therapeutics [21]. This review discusses the prospects and challenges of targeting the placenta using EV-based approaches as a therapeutic option to regulate insulin sensitivity in GDM and will initiate future deliberations in this direction.

## Insulin resistance in pregnancy and GDM

Insulin resistance is a characteristic feature of pregnancy. Insulin sensitivity decreases by 50–60% across gestation in healthy pregnancy and GDM [22,23]. However, when compared between the groups, GDM women have decreased insulin sensitivity compared with healthy matched controls [22,23]. Interestingly, the decrease in insulin sensitivity in GDM subjects compared to healthy women is evident even before conception and continues uniformly throughout gestation [22,23]. In addition, GDM women have increased hepatic insulin-stimulated glucose production compared with healthy pregnant controls, particularly in late gestation, which contributes to the hyperglycemia in GDM [22,23]. In healthy pregnant women, insulin secretion from the pancreas is increased in association with a decrease in insulin sensitivity. However, this compensatory mechanism is impaired in GDM, particularly in late gestation, which exacerbates the hyperglycemia leading to decreased oral glucose tolerance at the time of testing [22,24–26].

GDM is diagnosed at 24–28 weeks of gestation using an oral glucose tolerance test (OGTT) by giving 75 g oral glucose and, using the WHO2013 criteria, is considered present when one or more of the following criteria are met: fasting plasma glucose (≥5.1 mmol/L), 1 h (≥10.0 mmol/L), and 2 h (≥8.5 mmol/L) post 75-g oral glucose load [27]. However, the approach to GDM detection is not uniform across the world [28]. In certain countries a non-fasting glucose challenge test is performed to screen the women who require a full OGTT for GDM diagnosis [29,30]. Additionally, there is a lack of world-wide consensus regarding the threshold glucose levels for GDM diagnosis [28]. The risk factors for GDM include ethnicity, family history of Type 2 diabetes, previous history of GDM, overweight or obesity etc., and there is a wide variation in incidence levels of GDM in different countries [28].

Maternal hyperglycaemia in GDM leads to increased glucose influx to the fetal circulation causing fetal hyperglycemia. The excess glucose in fetal circulation leads to fetal hyperinsulinemia which causes fetal adiposity or macrosomia, the most serious complication of GDM [31]. GDM is also associated with maternal hypertensive disorders, dystocia, increased cesarean sections, and neonatal hypoglycemia [32,33]. Most importantly, GDM predisposes the women and offspring to a future risk of developing Type 2 diabetes, obesity, and cardiovascular diseases [34–37].

## Placenta and GDM

A healthy pregnancy outcome is highly reliant on tight physiological regulation that is largely orchestrated by an extremely complex and multifunctional maternofetal organ, the placenta [38]. The placenta plays a vital role in pregnancy, as it is involved in fulfilling numerous requirements of the growing fetus. It regulates the exchange of respiratory gases, provides protection for the fetus against maternal immunity, and removes carbon dioxide and excretions from the fetus via the mother. In addition, the placenta is a transient but very active endocrine organ, secreting various hormones and cytokines that can directly affect both maternal and fetal metabolism.

### Placenta in the metabolic regulation of pregnancy and GDM

Maternal insulin sensitivity during pregnancy is regulated by a complex interplay of several physiological factors which are not fully understood. However, factors or molecules secreted by the placenta have a pivotal role in reprogramming the maternal physiology throughout gestation. The placenta secretes several hormones such as placental growth hormone, placental lactogen, human chorionic gonadotropin and inflammatory mediators such as cytokines and leptin into the maternal circulation which can influence the maternal physiology and development of GDM.

Placental growth hormone has diabetogenic effects by suppressing insulin-stimulated glucose uptake and reduces maternal insulin sensitivity [39,40]. Specifically, placental growth hormone acts on the skeletal muscle and upregulates the expression of p85 which is the regulatory subunit of phosphatidyl inositol 3 kinase (PI3K), a key molecule in the insulin signaling pathway [39]. Excess p85α subunit competitively inhibits the docking of PI3K (p85-p110 heterodimer) to IRS-1 (insulin receptor substrate 1) in a dominant negative manner and impairs insulin signaling [41]. This leads to reduced glucose transporter activity and insulin resistance in skeletal muscle. Also, placental growth hormone is closely associated with the insulin-like growth factor (IGF) and correlates strongly with maternal glycemia and fetal weight [42]. Placental hormones such as placental lactogen, prolactin and human chorionic gonadotropin have a protective or antiapoptotic effect on pancreatic β cells and increases the β-cell proliferation and insulin secretion by various mechanisms [43–49].

Inflammatory mediators secreted by the placenta can influence insulin signaling and maternal metabolism in pregnancy [50]. Proinflammatory cytokines such as TNFα, leptin, and IL-6 are increased in the placenta from obese mothers implicating its crucial role in inflammatory changes in pregnancies [51–53]. Further, GDM is associated with increased expression of proinflammatory cytokines by the placenta [54,55]. Inflammatory pathways can directly impair the insulin signaling cascade and glucose transport, primarily by activating serine kinases and altering the phosphorylation of IRS-1 [56]. Kirwan et al., 2002, correlated the change in placental hormones, cortisol, leptin, and TNFα with the changes in insulin sensitivity and identified that TNFα is the most significant independent predictor of insulin sensitivity [57]. Plasma TNFα levels increase during late gestation, and this correlates negatively with insulin sensitivity [57].

### Placental dysfunction in GDM

The placenta supplies the fetus with all the necessary nutrients required for its growth and development and acts as a nutrient sensor, controlling maternal–fetal nutrient transport [58,59]. It detects maternal–fetal nutrient status and alters nutrient transporter capacity to align to fetal growth and nutrient requirements [60,61]. The metabolic dysregulation in GDM leads to excess glucose, amino acids, and fatty acids in the maternal system. The transfer of glucose across the placenta is concentration dependent and therefore in maternal hyperglycemia, excess glucose will be transferred to the fetus. Prolonged exposure of the fetus to high glucose and amino acids leads to fetal hyperinsulinemia. Further, aerobic respiration in fetal tissues is increased in the presence of hyperinsulinemia causing an increased oxygen demand in the fetal system. Consequently, the placenta increases its angiogenesis leading to placental hypervascularization as an adaptive response to facilitate oxygen diffusion to the fetus [62].

In addition, cholesterol synthesis in the placental endothelial cells is enhanced in GDM which increases the risk of atherosclerotic plaques in placental arteries. As an adaptive response, the placenta up-regulates its cholesterol efflux mechanisms and facilitates the removal of excess cholesterol from placental endothelial cells. All mechanisms to maintain cholesterol homeostasis are enhanced in the placenta to avoid formation of atherosclerotic plaques and vascular damage, thereby ensuring adequate blood flow for fetal development [62]. In this way, the placenta can sense changes in the maternal and fetal environment, which causes alterations in placental structure and function. However, in uncontrolled glycemia or abnormal lipid metabolism caused by pronounced obesity, placental homeostatic mechanisms become insufficient to provide adequate buffering, subsequently affecting the pregnancy outcome [62].

Maternal obesity and GDM alters the structure and molecular events in placenta with alterations in placental weight [63], gross morphology [64], transcriptome [65,66], proteome [67–70], lipidomic [71–74], and miRNA [75] profile. The effects of obesity and hyperglycemia driven changes in placental molecular landscape on the maternal and fetal outcomes has been reported [73,74,76]. For example, maternal obesity leads to decrease in uterine natural killer (NK) cells which in turn leads to alterations in the signaling pathways associated with extracellular matrix remodeling. This study implicates uterine immune cells as the link between defects in placental development and obesity [76]. Obesity associated inflammation can lead to changes in the molecular events in placenta with altered placental inflammation and oxidative stress [77]. Additionally, placental mitochondrial biogenesis pathway has be affected by maternal diabetic status and interestingly, the associated risk was higher in male fetus [78]. The insulin response in mother has been correlated to increase in placental weight which is more predominant in early pregnancy with influence on neonatal adiposity in male infants, and not in female infants [79].

**Figure 1:**
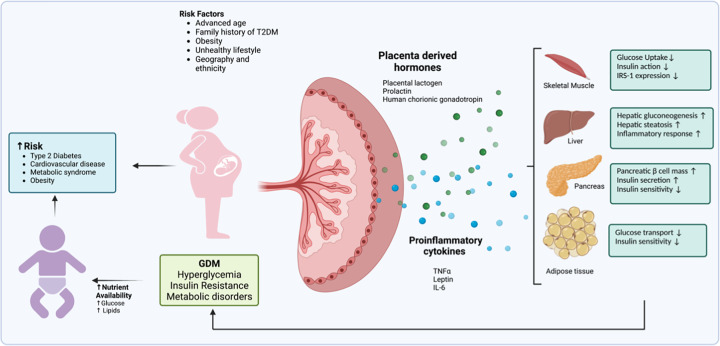
Placenta is central to metabolic regulation in pregnancy and mediates the short and long term consequences of GDM Several risk factors contribute to the development of GDM. Hormones and proinflammatory cytokines released from placenta regulate the insulin response in glucose homeostasis organs such as skeletal muscle, adipose tissue and liver, and insulin secreting pancreas and reprogram the maternal metabolism in healthy pregnancy and GDM. The metabolic derangement in GDM causes maternal hyperglycemia and the excess glucose and metabolites are transferred to fetus leading to short- and long-term consequences.

Prolonged exposure of the fetus to the altered metabolic intrauterine environment in GDM induces changes in the epigenetic reprogramming of the fetal genome leading to metabolic imprints with long-term effects on the offspring. Epigenetic changes altering gene expression profiles in association with metabolism in placental and fetal cells has been reported in GDM [80–85]. For example, placental DNA methylation is correlated with glycaemic levels in the mother during pregnancy [82,83]. Differential DNA methylation of the leptin gene in placenta in correlation to maternal OGTT glucose levels has been reported [83]. Placental tissue from GDM women has decreased methylation of MEST gene that encodes mesoderm-specific transcript homolog protein, involved in placental development and lipid metabolism [85]. A similar methylation pattern is reported in the fetal cells from GDM mothers implicating its long-lasting effects on the fetus [85]. Defective methylation of ABCA1 transporter gene responsible for cholesterol transport in cells has been detected in placental and fetal cells and correlates with dyslipidaemia in GDM mothers and fetus [80] Differential expression of placental miRNA expression has been reported and affects the placental function and pregnancy outcome [86–88]. Overall, GDM causes changes in the structure, function, and gene expression in the placenta which might be the result of adaptation of the placenta to an altered metabolic environment. Furthermore, placental dysregulation causes exposure of the fetus to a detrimental environment. This contributes to the explanation of why babies born to GDM mothers have an increased risk of developing obesity and Type 2 diabetes. The pathophysiology of GDM with placenta playing a central role as a mediator of short term and the long-term consequences of the metabolic changes is depicted in Figure 1.

## Extracellular vesicles and GDM

EVs were first described by Peter Wolf in blood coagulation studies using transmission electron microscopy and were described as cell waste [89]. However, substantial research in this area revealed that these membrane-derived vesicles interact with their target cells and modulate function via their biological signaling [19,90,91]. Following this discovery, EVs were isolated from various cell types such as platelets [92], hepatocytes [93], adipocytes [94], trophoblasts [95], and various cancer cells [96–100]. EVs have also been isolated from various bodily fluids such as plasma [101], bile [102], amniotic fluid [103], saliva [104], synovial fluid [105], breast milk [106], urine [107], and many others [108–111].

Although the generic term, EVs, is used to refer to the membrane bound vesicles, there are in fact, multiple subtypes of vesicles. EVs comprise a heterogeneous group of vesicles, classified on the basis of their origin, morphology, and mode of release into the extracellular milieu. These include microparticles, blebs, apoptotic bodies, and oncosomes [112]. Apoptotic bodies (0.8–5 µm in diameter) are released from cells undergoing programmed cell death [113]. Microparticles (0.1–0.35 µm in diameter), also known as ectosomes, originate from the external budding of the plasma membrane [114,115]. Small EVs (sEVs), also termed exosomes, are nano-sized vesicles (50–120 nm in diameter) formed from the inward budding of late endosomal structures called multivesicular bodies (MVB) and are exocytosed via fusion of MVBs with the plasma membrane [115,116]. The placenta is a rich source of EVs which includes all subtypes and various terminologies such as trophoblast debri, syncytiotrophoblast microparticles or microvesicles, syncytial aggregates etc., are used to describe them [117–119].

### Extracellular vesicles: biogenesis and heterogeneity

Small EVs called ‘exosomes’ are the most studied of all EV sub-types. The initial biogenesis and release of these endocytic nano-sized vesicles are the most critical steps in the EV signaling pathway, as this influences their biological functions and interaction with target cells. The biogenesis of exosomes begins with the formation of intraluminal vesicles (ILV) encapsulated in an MVB. The process of MVB docking and fusion on to the plasma membrane can occur via (i) endosomal sorting complex required for transport (ESCRT)-dependent mechanisms or (ii) non-ESCRT-dependent mechanisms.

The reorganization of the endosomal membrane by tetraspanins and recruitment of ESCRT are two essential components for the formation of ILVs. ESCRT consists of 20 proteins, assembled into four complexes, namely ESCRT-0, -I, -II, and –III, with associated proteins such as VPS4, VTA1, and ALIX [120,121]. The four ESCRT complexes coordinate to regulate vesicle budding and recruitment of the ubiquitinated proteins into the ILVs. ESCRT-0 is involved in membrane recruitment and endosomal capacity, and thus it recognizes the ubiquitinated proteins on the endosomal membrane and initiates the ESCRT pathway [122]. The recruitment of ESCRT-I by ESCRT-0 leads to activation of ESCRT-II by ESCRT-I [123]. ESCRT-I and –II drive the budding of the intraluminal membrane [124]. ESCRT-II recruits ESCRT-III that finally ends this process by mediating abscission of vesicles [125,126].

An ESCRT-independent pathway of EV biogenesis also exists in the absence of all four ESCRT complex subunits. This is shown by the formation of MVBs when ESCRT complexes are depleted in cells [127]. Another study demonstrated the formation of ILVs driven by sphingolipid ceramide independent of ESCRT complexes, and that exosome release decreases following inhibition of sphingomyelinases [128]. In this ceramide-dependent pathway, formation of lipid rafts prompts the formation of ILVs within the MVB [129].

EVs, when released into the extracellular space, can act locally but can also enter the circulation and cross physiological barriers, eliciting their actions at distant locations [130–132]. Surface molecules such as integrins can mediate selective targeting of exosomes to recipient cells [133–135]. Exosomes can interact with recipient cells by receptor–ligand interaction followed by uptake via phagocytosis or clathrin-coated endocytosis. Also, the exosomal membrane can directly fuse with the cell membrane and release their contents, or exosomes can initiate receptor mediated signaling.

Historically, there were inconsistencies in the naming of vesicles by EV researchers and EVs have been named according to their size, biogenesis, parent cell of origin, and disease condition with which they are associated [136]. This unregulated naming has created considerable confusion and lack of reproducibility for scientists working with EVs. Therefore, the International Society for Extracellular Vesicles (ISEV) laid down guidelines in 2018 to develop a consensus regarding EV nomenclature and improving reproducibility in the field. As per the ISEV2018 guidelines, an EV should be named as ‘small EVs’ for vesicles of size <100–200 nm in diameter and ‘medium/large EVs’ for vesicles of size >200 nm. Also, ISEV suggests the reporting of a minimum set of characteristic features that can distinguish an EV as either an exosome, microvesicle, or apoptotic body [137].

### Extracellular vesicles in GDM

A higher concentration of circulating EVs in maternal circulation is a characteristic feature of GDM [138,139]. In addition, alterations in the molecular cargo of sEVs [140–143] and bioactivity in the target cells have been reported in GDM [144–147]. Several studies have analysed the proteomic and miRNA content of maternal circulating EVs in GDM and NGT patients and evaluated the potential of the EV cargo as biomarkers and mediators of metabolic dysregulation [142–146]. Proteomic characterization of circulating sEVs from women at the time of GDM diagnosis (i.e., 22–28 weeks) revealed a specific set of proteins, which were differentially expressed in GDM [145]. This study identified protein pappalysin-1 (PAPP-A) to be down-regulated in circulating sEVs in GDM [145]. PAPP-A is a common biomarker used in early screening of pregnant women for pregnancy complications [148]. Another study reported the up-regulation of proteins associated with blood coagulation and lipid metabolism in GDM EVs [141].

Several studies performed the nucleic acid characterization of circulating EVs in GDM, miRNA being the most investigated cargo [140,142,143,149]. Next-generation sequencing revealed the comprehensive miRNA profile of EVs across gestation and the biomarker potential of the EV miRNAs for early prediction of GDM were evaluated [140,142]. We have identified that the miRNAs, miR-16-2-3p, miR-16-5p, miR-1910-5p, miR-423-5p, miR-92a-3p, and miR-92b-3p are differentially expressed in circulating EVs in GDM compared with NGT pregnancies. The expression profiles of these miRNAs were associated to the change in proteome in skeletal muscle and the biomarker potential of these miRNAs for the early detection of GDM at 18 weeks of gestation was evaluated [142]. In another study Ye et al., 2022, identified that miRNAs, miR-423–5p, miR-122–5p, miR-148a-3p, miR-192–5p, and miR-99a-5p were differentially expressed in GDM compared with NGT [143]. Further, they identified that these miRNAs target the IGF1R, GYS1, G6PC3, and FDFT1 genes and demonstrated their ability for early prediction of GDM at 10-16 gestational weeks [143]. Similarly, Thamotharan et al., 2022, identified the biomarker potential of EV miRNAs, miR-92a-3p, miR-192-5p, miR-451a, miR-122-5p, miR-92b-3p, miR-100-5p, and miR-125a-3p for the detection of GDM in the first trimester. Another study correlated the circulating EV levels of miR-222-3p and miR-409-3p to plasma fasting glucose levels indicating the potential link between EV miRNAs and insulin resistance in GDM [149]. However, there are fewer studies that analysed other species of RNA and DNA in circulating EVs with regard to GDM.

Molecular cargo in EVs has been extensively studied for their functional effects in target cells [138,139,142–144,147,150]. EVs are involved in the proliferation and migration of trophoblast cells, placental angiogenesis [139,151–154], immunomodulation in pregnancy [155], activation of inflammatory response [138,147,156], and regulation of insulin sensitivity in healthy pregnancy and GDM [142–144]. The role of sEVs in the regulation of maternal insulin sensitivity has been studied *in vivo*. Mice were infused continuously with sEVs derived from the plasma of GDM patients and it was shown that GDM sEVs induced glucose intolerance, and reduced insulin sensitivity *in vivo* compared with mice infused with sEVs from NGT patients [157,158]. The glucose intolerance was associated with changes in the miRNA profile, and insulin signaling in skeletal muscle tissues showed decreased phosphorylation of IRS-1 and Akt [157].

Fewer studies have reported the effects of EVs on pancreatic insulin secretion in healthy pregnancy and GDM. Analysis of the circulating EVs in GDM revealed a higher concentration of dipeptidyl peptidase-4 (DPP4) in placental EVs in GDM. These EVs had a higher level of DPP4 activity *in vitro* compared with EVs from NGT controls [159]. The DPP4 activity is intimately associated with pancreatic insulin secretion, and increased DPP4 activity causes cleavage and inactivation of the incretins, glucose-dependent insulinotropic peptide (GIP) and glucagon-like peptide 1 (GLP1), which cause decreased glucose-stimulated insulin secretion [160]. Also, infusion of sEVs from NGT pregnancies in mice models increased glucose-stimulated insulin secretion (GSIS) and fasting levels of insulin in pregnant mice compared to sEVs from GDM patients [157,158].

It is well established that high pre-pregnancy body mass index (BMI) or obesity confers an increased risk of developing GDM [161]. Interestingly, a positive correlation between the circulating sEV levels and maternal body mass index (BMI) across gestation was reported [156]. Obesity is associated with a pro-inflammatory environment, and sEVs isolated from obese women increased the release of pro-inflammatory cytokines from endothelial cells compared with sEVs from lean women [156]. As GDM and increased maternal BMI can influence the levels and bioactivity of sEVs during pregnancy [138,150,156], it is plausible to suggest a role for adipose tissue derived EVs in the regulation of maternal or placental metabolism, contributing to the development of GDM, especially in the setting of maternal obesity.

Interestingly, some studies have explored the fetal EVs in cord blood collected at the time of delivery and explored their relationship to the pathophysiology of GDM [162,163]. EVs derived from fetal endothelial cells in GDM are enriched in proteins involved in the oxidative stress response and linked to fetoplacental endothelial dysfunction [162–165]. A study by Zhu et al., 2023 reported differential profile of mRNAs in human umbilical cord endothelial cell derived EVs exposed to high glucose and identified that PUM2 (pumilio RNA binding family member 2) is highly enriched in EVs at high glucose. PUM2 regulates stability of SOX2 transcripts leading to translational repression and low SOX2 protein expression in trophoblast cells. PUM-mediated repression of SOX2 leads to defects in placental cell proliferation, invasion, and DNA damage there by mediating placental dysfunction in GDM associated pre-eclampsia [154]. The circular RNA profile in umbilical cord EVs has been analyzed, and shown as being associated with miRNAs that are altered in GDM [164]. Further, the mRNAs and lncRNAs in umbilical cord EVs have been analysed in GDM and identified that the differentially expressed mRNAs and lncRNAs in GDM are associated with the pathogenesis of GDM and fetal outcomes [165].

Placental alkaline phosphatase (PLAP) is a membrane protein present in the syncytiotrophoblast cells of the placenta, and is a specific marker for EVs of placental origin [117]. An analysis of PLAP +ve EVs in the maternal circulation identified that approximately 20% of the maternal circulating EVs are of placental origin [156]. GDM patients have higher levels of PLAP +ve EVs in circulation compared with NGT women [138]. Also, placental cultures such as primary cell cultures and explant cultures are reported to release higher concentrations of sEVs when obtained from GDM patients compared with NGT controls [138,139,144]. This indicates that the sEV biogenic machinery in placental cells might be responding to the pathological changes in GDM, manifest as an increase in sEV secretion. However, the underlying molecular mechanisms associated with this, which could be cell-intrinsic, environmental or both, are unclear. On the other hand, a microenvironmental control of sEV release from placental cells has been reported, in which hypoxia and high glucose can increase the release of sEVs from trophoblast cells [147]. Future investigations involving sEV biogenesis machinery and their components can lead to the development of attractive therapeutic targets in GDM.

**Figure 2:**
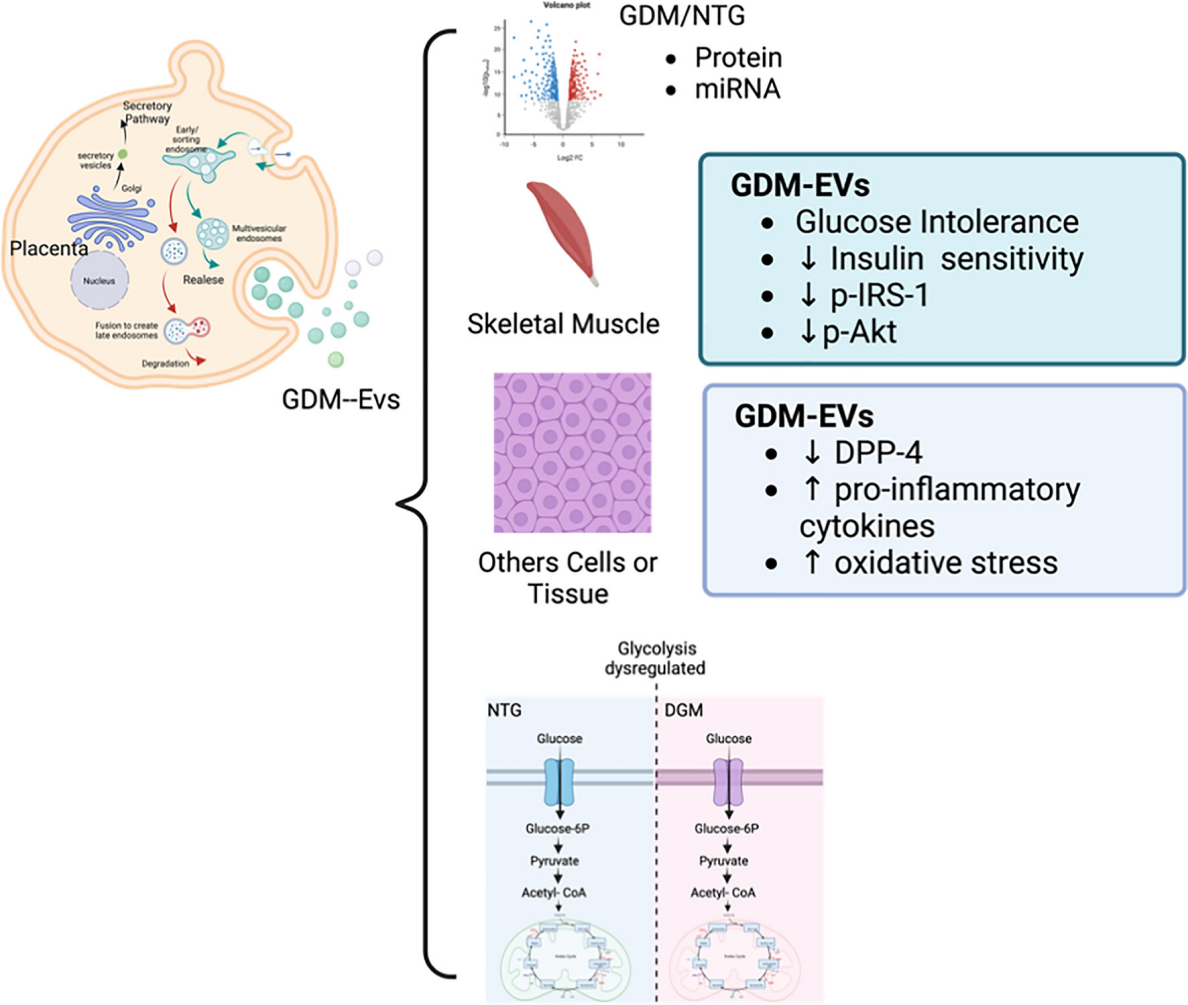
Placental EVs regulate insulin response and glucose homeostasis in target cells Placenta secretes a wide range of EVs into the maternal circulation. Placental EVs in GDM have differential expression of proteins and miRNAs compared with NGT EVs. GDM EVs induce glucose intolerance by decreasing the insulin sensitivity, phosphorylated IRS-1 and phosphorylated Akt expression in skeletal muscle. Placental EVs in GDM alter the DPP-4 activity in pancreas and increase the proinflammatory response and oxidative stress in different tissues. These changes lead to dysregulated glucose metabolism in cells and contribute to the metabolic derangement in GDM

Nevertheless, the contribution of placenta-derived EVs to the molecular cargo of total circulating EVs is unclear. Studies have identified a correlation between miRNA expression changes in the placenta in GDM and circulating EV miRNA expression [144,166]. We identified that miRNAs miR-125a-3p, miR-99b-5p, miR-197-3p, miR-22-3p, and miR-224-5p are differentially expressed in the placenta in GDM compared with NGT controls, and that this change in the miRNA profile is reflected in the circulating sEVs [144]. Importantly, placental sEVs from GDM patients can regulate the insulin-stimulated glucose uptake in skeletal muscle *in vitro*, highlighting the effect of sEVs in the regulation of maternal insulin sensitivity [144]. C19MC (Chromosome 19 miRNA cluster) is a miRNA cluster encoding 59 mature miRNAs and exclusively expressed by the placenta [167]. C19MC miRNAs are the most abundant miRNA species expressed in placental EVs [167] and are involved in placental maternal signaling [168]. Members of the C19MC such as miR-520h and miR-1323 were identified in circulating EVs in GDM [146]. Interestingly, altered expressions of C19MC miRNAs miR-516-5p, miR-517-3p, and miR-518-5p, were detected in urine EVs from GDM patients [169]. Taken together, the placenta plays a crucial role in the pathophysiology of GDM, and novel therapeutic strategies of targeting the placental factors in GDM is an exciting area of future research. The role of EVs in GDM and their effects on the insulin response and glucose metabolism in target tissue is illustrated in Figure 2.

## Targeting the placenta in GDM

The placenta holds an important role in the regulation of maternal metabolism. Regulating key molecules in the placenta associated with insulin resistance, using a targeting approach, can be a novel method of treatment in insulin resistance pregnancies. However, there have been limited investigations in this direction.

### Current strategies to target the placenta

Several studies have used nanoparticle delivery approaches to target the placenta in conditions of placental dysfunction to reverse the placental pathology [5,7,8,10,11]. For example, adenoviral vectors were used for the overexpression of vascular endothelial growth factor (VEGF) in maternal uteroplacental arteries, and the effect on placental function and fetal growth was studied [5,10,170]. VEGF is essential for angiogenesis and vascular development of the placenta [171]. Placental endothelial dysfunction is associated with decreased expression of VEGF in the utero placental arteries and associated with conditions such as pre-eclampsia [172]. Adenoviral vector containing the transgene for VEGF was delivered at the uterine arteries and transduced cell populations at the site of delivery. Also, intraplacental injection of adenovirus mediated IGF-1 has been developed as a strategy to correct placental insufficiency and improve fetal weight in intrauterine growth restriction (IUGR) murine and rabbit models [8,9]. Adenoviral vectors can deliver larger transgenes, but their DNA does not integrate with the host genome and resides episomally in the host nucleus. Hence, this mediates transient expression with no risk of mutagenesis, but the gene expression will be diluted amongst daughter cells with progressive cell division [173]. However, adenoviral-mediated gene therapy requires local injection of adenovirus vectors to the maternal uterine arteries [5,10] or the placenta [9].

On the other hand, systemic delivery of nonviral particles with a placenta targeting moiety on the surface, which is capable of delivering nucleic acids (small interfering RNAs, miRNAs, plasmids, etc.) or drug moieties is an attractive alternative. Zhang et al., 2018 used liposomes decorated with placental chondroitin sulfate A binding peptide targeting the glycosaminoglycan chondroitin sulfate A on expressed syncytiotrophoblast membrane [11]. The placental chondroitin sulfate A binding peptide is derived from a protein VAR2CSA, which is expressed on the surface of erythrocyte infected with the malarial parasite. Interestingly, chondroitin sulfate A is expressed by cancer cells and recombinant VAR2CSA protein binds efficiently to a wide range of cancer cells [174]. The placenta carries surface proteins specifically expressed in cancer cells, and typically absent in normal/non-cancerous tissues.

The placenta and cancer share similar developmental pathways and current knowledge about targeted therapeutics in cancer can be adopted for efficiently transferring cargo to the placenta. Tumor homing peptide (pentapeptide CGKRK and cyclic peptide iRGD) coated liposomes targeting the integrin receptors in placental vasculature have been used to deliver carboxyflourescein and IGF-2 to improve fetal weight in a fetal growth restriction mouse model [7]. Novel placental homing peptide CNKGLRNK coated liposomes targeting the placental and uterine endothelium has been used to selectively deliver nitric oxide donor to the placenta improving vasodilation in fetal growth restriction [6]. Studies that have targeted placenta to deliver therapeutic molecules and analyzed its effect on pregnancy outcomes are summarized in Table 1.

**Table 1:** Summarizing studies that have targeted placenta using specific targeting moiety in animal models and evaluated effects on pregnancy outcome

Targeting peptide	Ligand	Placental region/cell types targeted	Carriers	Therapeutic moiety	Animal model and route of administration	Effects on placenta, mother, or fetus	References
Placental chondroitin sulfate A binding peptide (PCSAbp)	Chondroitin sulfate A	Trophoblasts in mice and human placenta	Liposomes assembled from PLGA, lecithin and DSPE-PEG-COOH	Methotrexate	Healthy wild-type mice, i/v	Cytotoxic effect on placenta and fetus	[11]
			Nanoparticle constructed with carboxyl-polyethylene glycol-poly (d,l-lactide) (COOH-PEG5K-PLA8K), cationic lipid DOTAP	Nrf2 and sFlt-1 siRNA	Preeclampsia mouse model, i/v	Down-regulated expression of Nrf2 and sFlt-1 and increased expression of angiogenic factors in placenta	[145]
			PEG-PLA DOTAP nanoparticle	sFLT-1 siRNA	Healthy wild-type mice, i/v	Decreased sFLT1 mRNA level in placenta and protein level in serum	[146]
Tumor homing pentapeptide CGKRK	Calreticulin	Endothelial cells and trophoblast in the placental labyrinth, and within spiral arteries in mouse placenta and syncytiotrophoblast in human placenta	Iron oxide nanoworms and liposomes	Nil	Healthy wild-type mice, i/v	Reduced fetal weight in healthy wild mice	[7]
			Nil	miRNAs miR-145 and miR-675 inhibitor sequences	Healthy wild-type mice, i/v	Improved fetal and placental weights	[147]
Tumor homing cyclic peptide iRGD	Integrin α_v_	Endothelial cells and trophoblasts in mouse and human placenta	Iron oxide nanoworms and liposomes	IGF-2	Healthy wild-type and growth restricted mouse model i/v	Improved placental weight in healthy wild type mice, and increased fetal weight and weight distribution in growth restricted mice model	[7]
Placental homing peptide CNKGLRNK	Unknown	Endothelial cells in mouse placenta and uterus	Liposomes	Nitric oxide donor SE175	Healthy wild-type and endothelial nitric oxide synthase knockout (eNOS-/-) mice i/v	Increased fetal weight, decreased placental weight, increased spiral artery diameter and reduced placental oxidative stress in eNOS-/- mice	[6]

The placenta is a transient organ, hence ideal for targeting because there is rare possibility of adverse effect on the long term health of mother and offspring. Targeting the placenta can limit the amount of drug used. However, care should be taken to prevent any off-target effects on mother and fetus. Safety considerations are the most important when targeting the placenta, as the delivery molecules should not be transferred to the fetal circulation and cause adverse effects on fetal growth.

### EVs as a novel method for targeted therapeutics

Various studies suggest that EVs are promising delivery vehicles for transferring drugs and therapeutic molecules and have reported the uniqueness of EVs as drug delivery platforms [12,13]. EVs are extensively involved in cell communication through their ability to cross the endothelial lining of blood vessels and enter the circulation [175,176]. Typically, EVs can cross the blood–brain barrier [177,178], and are used as delivery vehicles to transfer drugs to brain cells in disease conditions [130]. However, the ability of EVs to cross the placental barrier is not clearly understood. The presence of maternal-EVs in fetal circulation has been detected [179], but the mechanism of transfer of EVs between the maternal and fetal compartments is unclear.

Interestingly, targeted biodistribution of EVs *in vivo* can be achieved by incorporating surface proteins in EVs, which can interact with target cell-specific proteins and assist in delivering cargo to specific recipient cells. There are several strategies to incorporate the cargo into EVs. One approach is to engineer donor cells to produce EVs with the desired surface targeting molecule and therapeutic biomolecule to be delivered to specific target cells [130,180–184]. The expression of targeting proteins by EVs can be achieved by cell engineering with plasmids that encode and overexpress the target protein, and fuse it with a membrane protein naturally incorporated in EVs during biogenesis [130,180,182–185]. For example, surface display of protein cargo can be achieved by fusing it to the transmembrane tetraspanin proteins, CD63 or CD81 [180,182,185]. Also, modifying the targeting protein by attaching myristoyl-group to the N-terminus [186] or glycosylphosphatidylinositol (GPI) to the targeting moiety [187] helps them to be preferentially incorporated into EVs. Natural EVs primarily accumulate in the liver, spleen, gastrointestinal tract, lungs, and kidneys of mice, depending on their cellular source and route of administration [188]. However, surface modification of EVs increases the circulation time of engineered EVs and inhibits their uptake and clearance by immune cells [189,190]. At the target cell level, the internalization and cargo delivery ability of engineered EVs can be enhanced by surface expression of vesicular stomatitis virus glycoprotein (VSVG) [191]. Cell engineering can be used to incorporate molecular cargo into EVs [192–195]. For example, RNA binding protein can be expressed and fused with EV membrane protein, CD9, and this leads to selective binding of miRNA and encapsulation in EVs [196].

Another approach is to modify EVs post-production by application of a targeting moity on the surface, and introduction of drugs or therapeutic molecules. Targeting peptides can be anchored on the EV surface by attaching to the phospholipids or other ligands on the EV surface [197], or by modifying the EV surface using different strategies such as covalent modification, namely click chemistry or non-covalent modification (electrostatic interaction, hydrophobic interaction, aptamer-based modification, etc.) [198–200]. Several methods such as electroporation, sonication, freeze–thaw cycles, and membrane permeabilization using chemical agents can be used to incorporate biological or chemical cargo in EVs [201–204].

**Figure 3:**
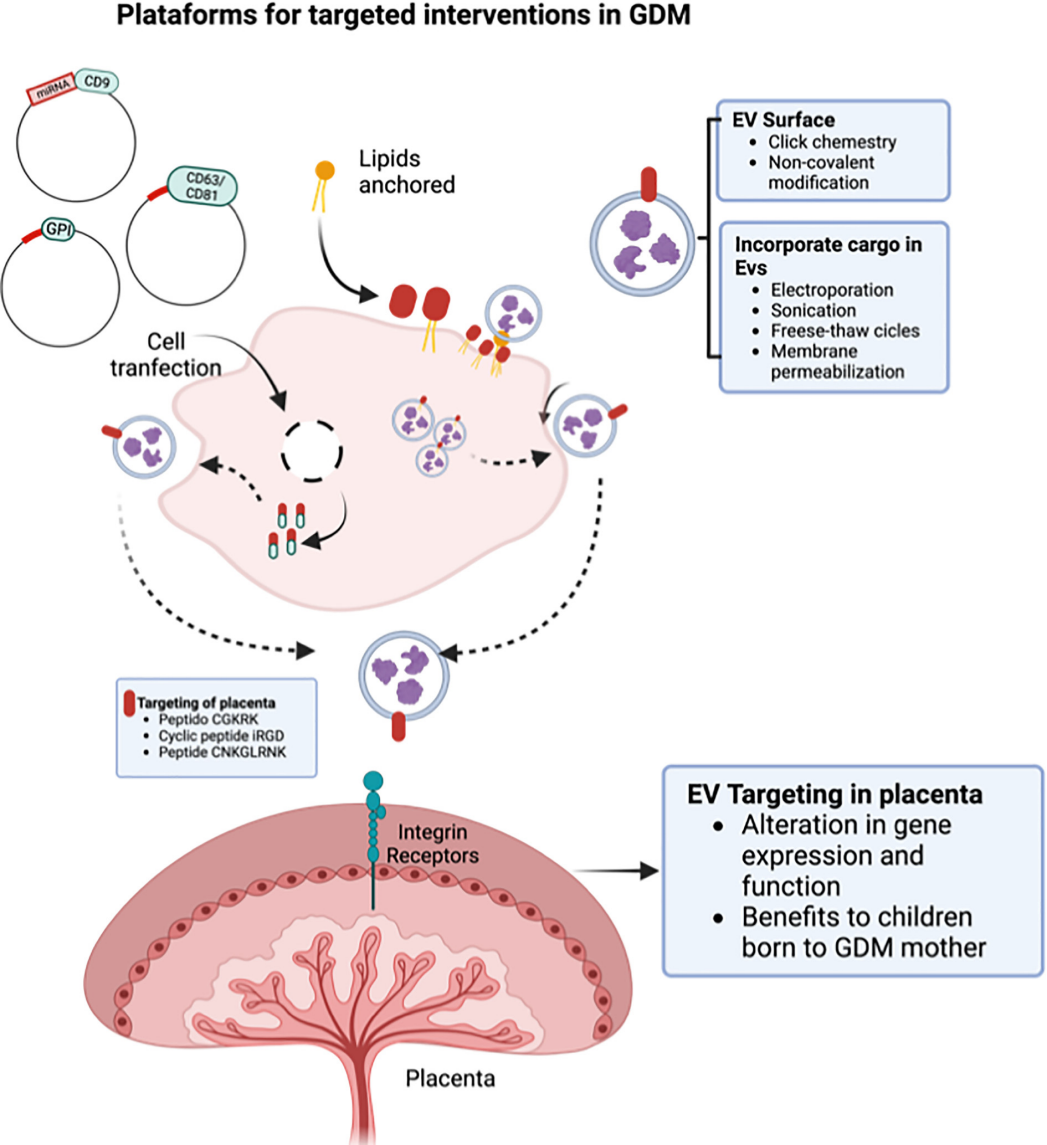
Engineering parent cells and EVs for targeted interventions in GDM EV-releasing cells could be engineered to produce EVs with surface expression of specific molecules that target placenta and could be loaded with therapeutic molecule. Also, natural EVs released from cells could be modified to express surface targeting molecule and therapeutic cargo incorporated using different methods to produce therapeutic EVs. These engineered EVs could be used for targeting placenta leading to changes in placental function and gene expression and leading to potential health benefits to women with GDM and their offspring.

Studies have reported the low toxicity and immunogenicity of EVs derived from human cell lines in preclinical models [205,206]. For example, in a study by Zhu et al., 2017, EVs derived from HEK293T cells were loaded with miRNA and protein and administered in C57BL/6 mice by intravenous and intraperitoneal routes for 3 weeks. On evaluation of the cytokine and immune cell profile, no signs of toxicity and minimal effects of immunogenicity were observed [205]. However, the immunologic and pharmacokinetic properties of EVs can vary depending on the parent cell of origin, mode of biogenesis and molecular content with limited studies reporting this [207]. These gaps in understanding the mechanism of EV signaling and the therapeutic efficacy, toxicity, clearance rate, and long-term effects of the administered EVs *in vivo* require future investigations. The potential EV-based platforms for targeted interventions in GDM is depicted in Figure 3.

### Challenges and future perspectives in EV based targeting approaches

EVs are naturally occurring particles with an intrinsic ability to cross barriers and deliver cargo, with minimal adverse effects and immunogenicity [130,205,208,209]. The possibility for exploiting cellular mechanisms to selectively load molecular cargo and perform surface modifications in EVs makes EVs versatile platforms for drug delivery.

Understanding of the biology of EVs, including their mechanisms of biogenesis and uptake at target cells is essential for designing engineering strategies for EV targeting. However, the diversity in the EV world poses a great challenge to understanding the basic biology of EVs, their mechanisms of origin, cargo sorting mechanisms, and interaction with the recipient cells. EVs comprise a diverse group of vesicles with multiple origins and overlapping physical and biochemical characteristics. This attributes considerable heterogeneity to the EV populations.

Another hurdle is the limitations in current EV isolation techniques to obtain pure populations of EVs, which is a major issue in studying EV biology and its applications in the clinical field [210,211]. The most common methods of EV isolation, such as centrifugation-based fractionation, size-exclusion chromatography, and ultrafiltration co-purifies proteins and other contaminants within EV populations [212–214]. Methods such as immune isolation have minimal contaminants but enrich only a specific sub-population of EVs. Flow cytometry based characterization of EVs allows characterization of heterogeneous mixtures of EV populations in bodily fluids [215]. The use of ISEV2018 guidelines for flow cytometry characterization of EVs allows for standardized reporting, and improved quality of studies from different laboratories. Furthermore, there is a paucity of understanding regarding the specific and unique marker proteins, which can specifically identify a particular subset or sub-population of EVs [216].

Analysis of EVs content has been done extensively to understand the functions of secreted EVs and to investigate their role in health and disease. However, there is a lack of specificity or reproducibility in profiling the molecular contents of EVs. The confusion attributed by the EV nomenclature and heterogeneity and the lack of standardization of the EV isolation protocols are the major contributing factors to this lack of specificity. In addition, different cell types secrete EVs with different molecular cargo, which is altered under different extracellular conditions such as pH and oxygen concentration, as well as concentration of different biomolecules in culture media [217,218]. Interestingly, there are specific sorting mechanisms in the cell, based on different RNA binding proteins (RBPs) that are associated with miRNA sorting in EVs, and this contributes to the biological heterogeneity in the EV cargo [219–221]. Hence, the variability from the cell culture should be minimized while manufacturing and scaling up EVs for therapeutic purposes. A multifactorial approach of fusing the EVs with nanoparticles to deliver the ‘best-of -both-worlds’ is an emerging area in nano medicine.

### Extracellular vesicle-based placental targeting future perspectives, and potential concerns

The placenta plays an important role as a mediator of insulin resistance by the release of hormones, cytokines and EVs that contribute to maternal metabolic regulation [2]. Placental hormones and inflammatory mediators such as TNFα can affect the phosphorylation status of insulin signaling molecules in key metabolic organs and directly contribute to the development of insulin resistance [39,57]. The EVs released by the placenta and the molecular cargo mediates cross talk between placenta and metabolic tissues, induces changes in insulin sensitivity [138,144]. In addition, metabolic derangement in GDM and obesity affects placental development and function and can have a long-lasting impact on neonatal phenotype [222]. Particularly, placental development is adversely affected by maternal metabolic and inflammatory changes. During placentation, expansion of the placental compartments responsible for nutrient transfer is regulated by the insulin and glucose levels in the maternal system, and defects in nutrient transfer is detrimental to the fetus [62,222]. There is a positive correlation between placental volume at first trimester and birth weight [223,224]. Similarly, maternal hyperglycaemia activates mitochondrial activity in placental cells and increases the release of reactive oxygen species resulting in placental dysfunction [225]. In maternal diabetes and obesity, there will be defects in spiral artery remodeling, which induces oxidative stress and placental maladaptation [226–228]. The placenta undergoes structural and functional changes as an adaptation to the altered metabolic milieu on maternal and fetal sides. The placenta strives to maintain a homeostatic environment optimal for the growth and development of the fetus. However, in poorly controlled maternal glycemia and obesity, there will be increased placental volume, inflammation and atherosclerosis in spiral arteries, changes in genes associated with metabolism, inflammation, coagulation, apoptosis and oxidative stress, and changes in miRNA profile and global increases in placental DNA methylation [62,222]. Nevertheless, the pivotal role of the placenta in the development of insulin resistance and determination of maternal and fetal outcomes opens an exciting avenue for targeted interventions in GDM.

The placental compartments involved in the multiple facets of GDM pathophysiology can be utilized as potential therapeutic platforms for targeted interventions in GDM. For example, targeting approaches modifying the placental release of factors/molecules regulating maternal insulin sensitivity could potentially improve glucose tolerance in GDM patients. The gene expression profiles associated with placental development, inflammation, metabolism, oxidative stress etc., could be selectively targeted to modify placental growth and vasculogenesis, maternal to foetal transfer of nutrients, placental genome imprinting, and methylation. As previously mentioned, the EV biogenesis pathway in trophoblasts respond to changes in maternal glycemia and oxidative stress and modify the levels and composition of released EVs [147]. However, therapeutic targeting of the EV biogenesis pathways can regulate EV release and molecular cargo and alter the placental signals to insulin target organs and facilitate metabolic adaptation. Specific genes knock down can be achieved by placental delivery of siRNA or antisense oligonucleotide or the CRISPR-Cas9 based genome editing system. On the other hand, over expression of specific genes can be achieved by delivery of plasmids expressing the transgene. EVs present an attractive option in targeting the placenta for efficient delivery of therapeutic biomolecules. EVs can be engineered with the therapeutic protein, RNA or CRISPR-Cas9 system, with a targeting ligand on the surface for site specific delivery to the placenta. EVs would protect the therapeutic molecules from degradation by nucleases and proteases in circulation, and fusion with the target cell will facilitate intracellular delivery. Surface modification of EVs with specific VSVG proteins enhances fusion with the target cell and preferentially delivers the cargo in the cellular endosomal compartments, and prevents trafficking to the lysosomal pathway [191]. Moreover, as EVs are from biological sources, they are biocompatible and their low toxicity and immunogenicity have been reported in preclinical [205,206] and clinical models [208,209]. EVs have a prolonged life span in blood circulation, or low clearance rate in blood, and the possibility of engineering the donor cells to synthesize EVs with desired characteristics helps them outperform synthetic nanoparticles.

However, there should be further studies to understand the EV biology, synthesis, and uptake, which will improve our expertise in engineering EVs with desired properties. Care should be taken to ensure that the targeted therapeutics will deliver the molecule specifically to the placenta without off target effects to the other maternal and fetal tissues. Even though the placenta is a transient organ removed at the time of delivery, changes in placental gene expression can have long-lasting effects on the fetus. Also, there is a gap in understanding of the mechanisms by which molecules are transferred across the complex barrier of the placenta. Hence the possibility of targeting molecules being transferred to the fetal circulation and causing off target effects on fetal tissues could be a potential concern. Overall, EV-based placental targeting in GDM is a novel and attractive therapeutic strategy to prevent the transgenerational transfer of metabolic disorder and offer long term health benefits to the offspring.

## Conclusion

Metabolic derangement and excessive insulin resistance without sufficient compensation by increased insulin secretion in GDM predisposes the mother and offspring to both pregnancy complications and future risks of developing metabolic disorders and obesity. The placenta has a key role in the regulation of maternal metabolism by reprogramming insulin sensitivity in healthy pregnancy, and GDM. Also, the placenta undergoes adaptive changes to maternal metabolic events and derangement in placental structure and function results in maternal diabetes and obesity. Hence, therapeutically targeting placental mechanisms involved in GDM pathophysiology offers a novel and interesting approach. EVs being biological carries of signaling molecules could potentially be engineered to deliver therapeutic biomolecules to specific cells in the placenta. EV-mediated targeting approaches could potentially cause specific targeted alterations in the gene expression and function of the placenta and offer long-term health benefits to children born to GDM mothers. Developing strategies to target the placenta during pregnancy is vital for improving maternal health, preventing complications, and optimizing fetal development. A well-functioning placenta ensures proper nutrient exchange and hormonal regulation, impacting both short- and long-term outcomes for both mother and baby. Targeted interventions offer potential advances in reproductive health. Future studies successfully integrating the knowledge in EV biology with placental mechanisms in GDM and other pregnancy complications, and expertise in therapeutic targeting can open up new pathways to treating metabolic diseases.

## Data Availability

Not applicable.

## Open Access

Open access for this article was enabled by the participation of University of Queensland in an all-inclusive *Read & Publish* agreement with Portland Press and the Biochemical Society under a transformative agreement with CAUL.

## Abbreviations

DPP4: dipeptidyl peptidase-4
EV: extracellular vesicle
GIP: glucose-dependent insulinotropic peptide
GLP1: glucagon-like peptide 1
GPI: glycosylphosphatidylinositol
GSIS: glucose-stimulated insulin secretion
IUGR: intrauterine growth restriction
OGTT: oral glucose tolerance test
PLAP: placental alkaline phosphatase
VEGF: vascular endothelial growth factor
VSVG: vesicular stomatitis virus glycoprotein
